# Malignant Arrhythmic Mitral Valve Prolapse: A Continuum of Clinical Challenges from Diagnosis to Risk Stratification and Patient Management

**DOI:** 10.3390/jcdd8010002

**Published:** 2020-12-29

**Authors:** Idit Yedidya, Aniek L. van Wijngaarden, Nina Ajmone Marsan

**Affiliations:** 1 Department of Cardiology, Leiden University Medical Center, Leiden, 2300RC Zuid-Holland, The Netherlands; I.yedidya@lumc.nl (I.Y.); A.L.van_Wijngaarden@lumc.nl (A.L.v.W.); 2 Department of Cardiology, Rabin Medical Center, Petah-Tikva 49372, Israel

**Keywords:** mitral valve prolapse, sudden cardiac death, risk factors

## Abstract

Mitral valve prolapse (MVP) is a common valvular disease, which may remain a benign condition for a long period of time. However, some patients experience malignant ventricular arrhythmias and sudden cardiac death (SCD). It is still largely unknown how to risk-stratify these patients, and no specific recommendations have been proposed to help the clinical decision-making. We present the case of a young man whose first clinical presentation was an out-of-hospital cardiac arrest and was subsequently diagnosed with MVP. We highlighted the possible risk factors for SCD and the challenges in the clinical management of these patients.

## 1. Introduction

Mitral valve prolapse (MVP) is one of the most common valvular heart diseases, which may remain a benign condition for a long period of time, unless accompanied by severe mitral regurgitation (MR) which requires surgical treatment when patients develop symptoms or the left ventricle (LV) begins to dilate and dysfunction. However, some patients, usually young and without any significant comorbidities, experience malignant ventricular arrhythmias (VA) and sudden cardiac death (SCD), estimated in 0.2–0.4% per year, and up to 1.8% per year in the presence of a flail leaflet [1,2]. Several studies have identified some risk factors associated with malignant arrhythmias and SCD in MVP patients, mainly (i) female sex, (ii) bi-leaflet prolapse, (iii) mitral annulus abnormalities such as dilatation and disjunction (defined as a separation between the left atrial (LA) wall at the level of the valve junction with the LV free wall), (iv) electrocardiographic repolarization abnormalities specifically on the inferolateral leads [3,4,5], (v) frequent and complex premature ventricular contractions (PVCs), and (vi) presence of myocardial fibrosis at the level of the papillary muscles [6,7]. However, it is still largely unknown how to risk-stratify these patients and no specific recommendations have been proposed to help the clinical decision-making.

We present the case of a young man whose first clinical presentation was an out-of-hospital cardiac arrest and was subsequently diagnosed with MVP, with the aim of highlighting the diagnostic tests performed to exclude other causes of SCD and to identify possible specific risk factors in MVP, and of creating further awareness of the challenges in the clinical management of these patients.

## 2. Case Report

A 44-year-old man was admitted to the hospital after an out-of-hospital cardiac arrest, with a successful resuscitation including nine shocks for ventricular tachycardia/ventricular fibrillation (VT/VF). Retrospectively, it was discovered that he had hypertension and hyperlipidemia and had been treated with an angiotensin-converting enzyme inhibitor and a statin. His family medical history included early coronary heart disease (father’s side) and SCD in his mother’s family, which was never further explored. Before the event, he had no specific cardiac complaints.

**Figure 1:**
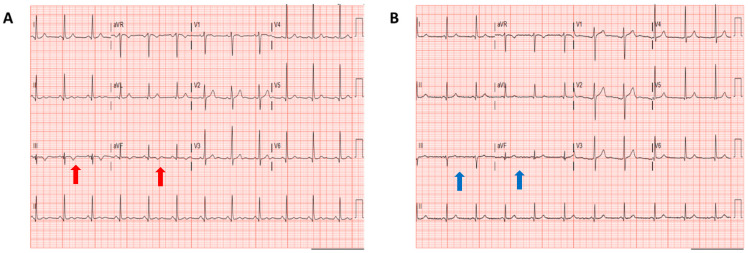
Comparison of electrocardiogram (ECG) before and after mitral valve repair. Panel (**A**): ECG during hospitalization, showing T wave inversion on lead III, AVF (red arrows). Panel (**B**): ECG after mitral valve repair, showing normalization of the T wave changes (blue arrows).

**Figure 2:**
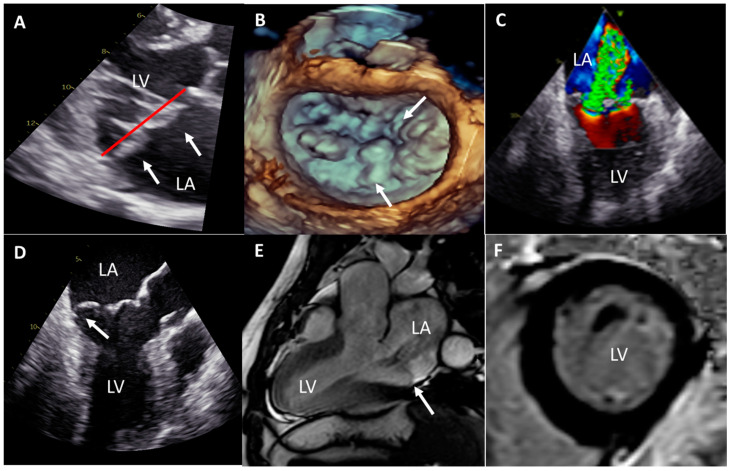
Imaging of the mitral valve prolapse and of the left ventricle. (**A**)Trans-thoracic echocardiography, parasternal long-axis view, demonstrating thickened bi-leaflet prolapse (white arrows) of the mitral valve. The red line identifies the mitral annular level. LA: left atrium; LV: left ventricle. (**B**) Trans-esophageal 3D echocardiography of the mitral valve, showing the detailed anatomy of the multi-scallop, bi-leaflet prolapse (white arrows) of the Barlow′s-like mitral valve. (**C**) Trans-esophageal Color Doppler echocardiography (bi-commissural view), showing the severe (multi-jet) mitral regurgitation. (**D**) Trans-esophageal echocardiography (three-chamber view) showing the prolapse of the mitral valve posterior leaflet and mitral annular disjunction (MAD) (white arrow). (**E**) CMR (three-chamber view cine) showing the prolapse of the mitral valve with mild MAD and dilated LV. CMR: cardiac magnetic resonance. (**F**) CMR (delayed enhancement scan of one representative slice of the short axis at the level of the papillary muscles), showing no evidence of macroscopic myocardial fibrosis/scar (black myocardium without any white signal).

Upon arrival to the emergency room, he was hemodynamically unstable with a blood pressure of 80/50 mmHg, and on physical examination, a grade II/III systolic murmur was noted at the apex. The electrocardiogram (ECG) showed a junctional rhythm without signs of acute coronary syndrome, but the patient was brought immediately to the catheterization-laboratory, where no significant coronary artery disease was found, and a temporarily LV support device was positioned (Impella, Abiomed). The blood tests did not show any abnormalities except a peak troponin T of 1392 ng/L. After hemodynamic stabilization, the LV support device was removed, and at the fourth day of hospitalization he was weaned from ventilation support. The ECG then showed a sinus rhythm with T wave inversion on lead III and AVF (Figure 1); at the telemetry, several PVCs could be observed but without non-sustained VT. A complete echocardiogram was performed, which showed a dilated LV (end-diastolic diameter = 63 mm) with normal systolic function, a moderate-severe late-systolic MR by a bi-leaflet prolapse (Barlow’s MVP) with a mild mitral annulus disjunction (<5 mm) but no evidence of flail or rupture chordae (Figure 2), enlarged LA (volume index = 61 mL/m^2^), and right ventricle systolic pressure within normal range.

A contrast-enhanced cardiac magnetic resonance (CMR) imaging was performed on the eighth day of admission to exclude cardiomyopathy, and showed a dilated LV (end-diastolic volume index = 137 mL/m^2^) with no regional wall motion abnormalities, good systolic function (ejection fraction = 56%) and no presence of myocardial fibrosis; the presence of the bi-leaflet prolapse was confirmed with mild mitral annular disjunction (Figure 2). The lack of late gadolinium enhancement (myocardial scar or fibrosis) on CMR, together with the absence of regional wall motion abnormalities, of significant coronary artery disease by coronary angiography and of dynamic ECG abnormalities, confirmed the exclusion of ischemic heart disease as a cause for the cardiac arrest.

Considering the out-of-hospital cardiac arrest without clear reversible cause, an implantable cardioverter defibrillator (ICD) was implanted, and the patient was discharged. Before discharge, genetic counseling was performed according to standard practice including whole exome sequencing. Firstly, 522 genes associated with cardiovascular development and disease were evaluated, with special attention to the genes know to be associated with mitral valve prolapse (Dachsous Cadherin-Related 1(DCHS1), Filamin A (FLNA), Phospholipase D1 (PLD1), and DAZ interacting protein 1(DZIP1) [7]. Furthermore, family screening was advised, and his sister also underwent echocardiography (which revealed also a Barlow’s MVP with mild MR) and genetic testing. So far, no known genetic mutations have been found, but the comparison of the whole exome analysis of the brother and sister is still ongoing to try to find new pathogenic variants.

During the out-patient clinical follow-up, two months after discharge, a Holter recording was performed and showed sinus rhythm with no complex arrhythmias and 1% PVCs. A few months later, the patient developed mild symptoms (reduced exercise capacity), and based on the presence of LV dilatation and significant MR, he was referred for mitral valve repair and concomitant tricuspid valve repair (based on a tricuspid annuls of 40 mm and tricuspid regurgitation grade 2+). In particular, mitral ring annuloplasty was performed (Edwards Physio-II ring 38) and two pairs of neochordae were placed on P2. Tricuspid valve ring annuloplasty was also performed (Edwards Physio Tricuspid ring 32). The surgical operation was successful with no residual mitral regurgitation, and an echocardiogram six months after the operation showed normalization of the LV dimension with good systolic function. The ECG abnormalities also disappeared with normalization of the negative T waves (Figure 1). The patient remained asymptomatic with no ICD shocks until now.

## 3. Discussion

### 3.1. Risk Stratification for Ventricular Arrhythmias in MVP Patients

Identification of MVP patients at risk of malignant arrhythmias is still a major clinical challenge. Studies published so far on the topic have been performed retrospectively, and mainly include a selected population based on the outcome of SCD [2,3,6]; therefore, the arrhythmic phenotype of MVP is far from being properly defined. In a study of patients experiencing SCD, patients with MVP tended to be younger and with less traditional cardiovascular risk factors for SCD such as diabetes mellitus and hypertension, as compared to other patients [8]. However, some risk factors have been proposed, including female sex, bi-leaflet prolapse, mitral annulus dilatation and disjunction, T wave inversion in the inferolateral leads, frequent and complex PVCs, and presence of papillary muscle fibrosis [9]. The combination of these parameters suggests that in these patients VA may be the result of a valvular trigger acting on a pathological myocardial substrate.

In the patient of the current case, extensive analysis has been performed to exclude all possible causes of SCD and identify the proposed risk factors for malignant VA in MVP; however, the patient presented few of the proposed risk factors that might have helped in refining his risk profile. At the first echocardiographic analysis, a clear Barlow’s-like valve with a bi-leaflet prolapse and a significant annular dilatation and valvular regurgitation was observed, but the mitral annulus disjunction was only mild (<5 mm). Mitral annulus disjunction has been proposed to be associated with VA, but particularly when ≥5 mm, and in patients without significant MR [10]. Our patient also presented, soon after hospitalization and until operation, T wave inversion in the inferior ECG leads, which was reported as highly prevalent in patients with MVP and cardiac arrest [3], and is strongly associated with VA [7]. Interestingly, these ECG abnormalities disappeared soon after mitral valve repair, suggesting that LV volume overload reduction or the relief of the mitral valve apparatus stretching after operation can normalize these ECG changes, as also reported by Alqarawi et al [11].

Advanced imaging such as CMR, has been proposed to better characterize the myocardial substrate in these patients and particularly to identify the presence of late gadolinium enhancement (LGE) at the level of the papillary muscles or basal inferolateral LV wall, which have been associated with VA. In the study by Basso et al [10], 93% of patients with MVP and complex arrythmia showed LGE in the papillary muscle and LV inferonasal wall, while Dejgaard et al showed that 46% of patients with mitral annuls disjunction and severe arrhythmogenic events had LGE on CMR [10]. Our patient showed no LGE on CMR, but had a dilated LV; however, assessment of diffuse myocardial fibrosis by T1 mapping imaging was not performed.

### 3.2. How to Improve Risk Stratification for Ventricular Arrhythmias in MVP Patients

Initial studies have tried to identify novel markers to refine risk stratification for VA in these patients. On top of the above-mentioned MV abnormalities, advanced echocardiographic measures such as global longitudinal strain (GLS), post-systolic index and mechanical dispersion were found to be associated with VA [9], and might help physicians in referring these patients for further analysis such as CMR. Due to the high prevalence of MVP and benign outcomes [12], a selection of patients at higher risk who should be referred for more advanced and expensive examinations are required. CMR can better quantify MR and LV dilatation and characterize the presence of mitral annular disjunction, but is especially indicated to identify myocardial focal fibrosis which was described in these patients at the level of the papillary muscles or at the basal LV adjacent to the MV, as a result of the increased mechanical traction by the prolapsing valve [13]. However, previous observations suggested that the myocardial substrate for VA might also be represented by a diffuse myopathy rather than only focal myocardial alterations, and initial studies have shown an increased myocardial interstitial fibrosis in these patients using CMR T1 mapping [14,15].

In our patient, a potential point of clinical attention was the family history of SCD which was not further investigated before the event. A screening including echocardiography and Holter could have been indicated at the time of death of his mother. Similarly, when the diagnosis of MVP is made in a patient, thorough family anamnesis for valvular heart disease and SCD should be performed (which is currently only typically asked for in patients with coronary artery disease) and a Holter recording may be considered even without symptoms. More importantly, family screening should be advised, as for our patient whose sister was also consequently diagnosed with MVP. A familial distribution of MVP has been largely documented especially in Barlow’s patients [16,17] and four genes are known so far to be linked to MVP; interestingly, also an association of isolated MVP and mutations in cardiomyopathy genes was observed, further supporting the important role of myocardial involvement in the disease [18,19]. Finally, the possibility of testing specific biomarkers might be offered in the future; for example, initial observations showed the soluble suppression of tumourigenicity-2-serum level (sST2), as a marker of the stretched myocardium, to be higher in patients with mitral annular disjunction and VA [20].

### 3.3. Therapeutic Options for Ventricular Arrhythmias in MVP Patients

Due to a lack of evidence, current guidelines unfortunately cannot provide specific indications for the treatment of VA in these patients, but only management advice according to known risk factors for other cardiovascular disease. Medical therapy should be the same as for a non-MVP population, with a potential use of catheter-based ablation in the case of high burden and symptomatic arrhythmias [21]. ICD implantation, as for the non-valvular population, is currently indicated only for secondary prevention (as in our patient) or in the case of severely depressed LV function. Although MV repair is currently indicated only based on the severity of MR and LV characteristics, initial studies have shown a lower incidence of VA, including less ICD-appropriate shocks, after surgery for MVP [22,23]. In our patient, after mitral valve repair surgery (indicated because of symptomatic severe MR), we found no remaining evidence of complex arrhythmias, including no PVCs, and normalization of the ECG abnormalities and LV dimension.

In conclusion, the current case aims at creating further awareness on the comprehensive diagnostic analysis (including genetic testing) necessary when a patient presents with SCD and MVP as the only possible cause, and the challenges a cardiologist faces in the management and decision-making of these patients due to the lack of specific recommendations.

## Data Availability

Data available on request due to restrictions of privacy.
